# Large litters have a detrimental impact on litter performance and postpartum maternal behaviour in primiparous sows

**DOI:** 10.1186/s40813-024-00360-2

**Published:** 2024-02-16

**Authors:** Juho Lee, Hyeonwook Shin, Junsik Kim, Geonil Lee, Jinhyeon Yun

**Affiliations:** Department of Animal Science, College of Agriculture and Life Sciences, Chonnam National University, 61186 Gwangju, South Korea

**Keywords:** Hyperprolific sows, Loose housing, Nursing behaviour, Oxidative stress, Salivary cortisol

## Abstract

**Background:**

Our previous study confirmed that large litter size adversely affects prepartum maternal hormones and behaviour, concurrently with heightened oxidative stress in primiparous sows. The purpose of this study was to examine the effect of large litter size on litter performance, postpartum maternal behaviour, salivary cortisol levels, and colostral immunoglobulin levels in sows, as well as investigate their correlations with the levels of oxidative stress parameters.

**Results:**

A total of 24 primiparous sows (Landrace$\times$Large white) and their offspring were categorised into two groups based on litter size: NORMAL (*n* = 8) with litter size ranging from 7 to 14 (mean 11.5$\pm$2.7), and LARGE (*n*=16) with litter size ranging from 15 to 20 (mean 15.9$\pm$1.4). All sows were housed in a group housing system during gestation and transitioned to an adaptable loose housing system (2.4$\times$2.3 m) during the farrowing and lactation periods. The nursing and carefulness behaviour of the sows was monitored over a 24-h period between 72 and 96 h after parturition. Saliva samples were collected for cortisol assay on 35, 21, and 7 days before parturition (D-35, D-21, and D-7, respectively), as well as on days 1, 7, and 28 after parturition (D1, D7, and D28, respectively). On D1, higher piglet mortality rates were observed among the LARGE group compared to the NORMAL group (*p*<0.01). The total and successful nursing behaviours of the sows were less frequent in the LARGE group than in the NORMAL group (*p*<0.05, for both), and the carefulness score of the LARGE group was also lower than that of the NORMAL group (*p*< 0.01). On D1, cortisol levels in LARGE sows were higher than those in NORMAL sows (*p*< 0.05), and for other time points (D-21, D-7, D7, and D28), cortisol levels in LARGE sows tended to be higher than those in NORMAL sows (*p* < 0.10, for all). Successful nursing behaviour displayed negative correlations with levels of salivary cortisol and certain oxidative stress parameters measured on D1.

**Conclusions:**

These findings suggest that the strategy for alleviating physiological and oxidative stress during the peripartum periods could benefit potential postpartum maternal behaviour and litter performance in the sows with large litters.

## Background

In the past three decades, genetic selection for larger litter sizes in the pig industry has led to a significant rise in the number of piglets born alive, increasing from 10 to 20 per litter [1]. However, this increase in litter size has been accompanied by a simultaneous decrease in the birth weight of piglets and an extension of the farrowing duration from 1.5 to 2 to 7–8 h [1, 2].

Studies have reported that low birth weight in piglets and prolonged farrowing are associated with reduced neonatal piglet vitality [3]. Low-vitality piglets not only require more time to initiate their first teat sucking, but they also exhibit limited competitiveness when competing for teats among their littermates [4], leading to a decrease in their milk intake [2]. Moreover, hyperprolific sows often face a shortage of functional teats to nurse their large litters [2]. These challenges contribute to a high preweaning piglet mortality rate in hyperprolific sows [2]. Therefore, given these challenges and findings, there is a pressing need for research aimed at improving lactation performance and maternal behaviour in hyperprolific sows to reduce piglet mortality rates.

Prolactin and oxytocin, which are maternal hormones secreted by the pituitary gland, play a vital role in influencing lactation performance and maternal behaviour in sows [5–7]. However, the secretion of these hormones can be inhibited by physiological stress, which is associated with an increase in opioid receptors [8–11]. Previous research has indicated that hyperprolific sows, in particular, are susceptible to experiencing substantial stress owing to various factors, such as multiple foetal growths, prolonged farrowing, and the high demands of milk production from late gestation through lactation [12]. Furthermore, our recent companion study revealed that hyperprolific sows exhibited elevated levels of oxidative stress during late gestation and lactation compared to normally prolific sows [13]. This study has also raised the possibility of a potential association between elevated oxidative stress and reduced prolactin levels [13].

The acquisition of passive immunity through colostral immunoglobulins is crucial for piglet survival. This is because the epitheliochorial placenta impedes the transfer of maternal immunoglobulins to the developing foetuses during pregnancy [14]. The majority of colostral IgG and IgM, as well as 40% of IgA in sows, are derived from their bloodstream [15]. However, previous studies have reported that physical stress can decrease the serum immunoglobulin levels in sows [16]. Similarly, oxidative stress has been found to lower serum immunoglobulin levels in rats as well [17].

Given this context, enhanced knowledges regarding the negative influence of large litter sizes on the physiological status of sows is crucial for improving litter performance. Therefore, our primary objective was to evaluate the impact of an increased litter size at birth, which can potentially induce oxidative stress, on postpartum maternal behaviour, salivary cortisol levels, and litter performance in primiparous sows. We hypothesised that an increased litter size could have a negative influence on postpartum maternal behaviours, possibly due to heightened physiological and oxidative stress levels in peripartum sows. To investigate this hypothesis, we examined the potential correlations between nursing and carefulness behaviors observed three days after parturition. Additionally, we explored the associations with peripartum salivary cortisol levels and oxidative stress statuses of the primiparous sows. We used data on salivary oxidative stress parameters and colostrum oxytocin and prolactin from our previous publication by Lee et al. [13]. We anticipated that postpartum maternal behaviour, salivary cortisol levels, litter performance, and colostral immunoglobulin levels would differ between sows with normal and large litter sizes, based on their respective oxidative stress statuses.

## Results

### Piglet mortality and growth

On D1, the piglet mortality rate was greater in the sows in LARGE group compared to those in the NORMAL group (*P* < 0.01, Table 1). Furthermore, the piglet mortality rate due to crushing on D1 tended to be greater in the LARGE group compared to the NORMAL group (*P* = 0.05, Table 1). The mortality rate due to other causes on D1 was also greater in the LARGE group compared to the NORMAL group (*P* < 0.05, Table 1).

**Table 1:** Comparison of litter performance between the normal litter (NORMAL) and large litter (LARGE) groups

N	NORMAL	LARGE	SEM	*P* value
	8	16		
Piglet mortality (%)				
D1^1^	1.3	4.0	1.0	< 0.01
Crushing	1.1	2.5	0.8	0.053
Other causes	0.2	1.5	0.1	0.013
D7^2^	1.9	7.2	1.5	0.103
D28^2^	2.9	7.7	1.5	0.137
Piglet body weight (kg)				
D1^1^	1.7	1.4	0.1	0.033
D7^2^	3.0	2.6	0.1	0.037
D28^2^	7.6	7.3	0.2	0.338

^1^ The number of nursery piglets before cross-fostering in the NORMAL and LARGE groups was 11.4 ± 1.2 and 15.4 ± 0.4, respectively

^2^ Cross-fostering was performed within 48 h after the end of parturition, and the litter size was standardized to 12.6 ± 2.3 piglets per sow

On D7, the piglet mortality rate in the LARGE group tended to be greater than that in the NORMAL group (*P* = 0.10, Table 1). However, there were no significant differences in the piglet mortality rate on D28.

The piglet body weights in the LARGE group were lower on D1 and D7 than those in the NORMAL group (*P* < 0.05, for both, Table 1). However, there were no differences in piglet body weights between the sow groups on D28 (Table 1).

### Nursing and carefulness behaviour on D3 postpartum

Sows in the LARGE group exhibited reduced total and successful nursing bouts compared to those in the NORMAL group (*P* < 0.05 for both, Table 2). The duration of total and successful nursing was longer for sows in the LARGE group compared to those in the NORMAL group (*P* < 0.01 for both, Table 2). The sow-terminated nursing rate was significantly higher in the LARGE group compared to the NORMAL group (*P* < 0.05, Table 2).

**Table 2:** Comparison of nursing behaviour, carefulness score, and lying-down events between sows with normal litters (NORMAL) and large litters (LARGE)^1^

N	NORMAL	LARGE	SEM	*P* value
	8	16		
Total nursing behaviour				
Frequency	35.8	30.7	1.1	0.035
Duration (min)	232.4	180.4	8.4	< 0.01
Bout duration (min)^2^	6.5	5.9	0.2	0.115
Sow-terminated nursing, (%)	25.5	40.2	3.5	0.039
Successful nursing behaviour				
Frequency	23.8	19.5	0.8	0.033
Duration (min)	162.1	122.1	6.8	< 0.01
Bout duration (min) ^2^	6.8	6.3	0.2	0.344
Carefulness score (0–5)^3^	3.4	2.8	0.1	< 0.01
Lying-down event (n)	8.7	8.5	1.4	0.901

^1^ Behaviours were monitored from 72 to 96 h after the end of parturition

^2^ Duration/frequency

^3^ Represents the total occurrence [1] or non-occurrence (0) of each carefulness behaviour parameters

The frequency of lying down events showed no significant differences between the two groups. However, the LARGE group exhibited a lower carefulness score than the NORMAL group (*P* < 0.01, Table 2).

### Salivary cortisol levels

**Fig. 1:**
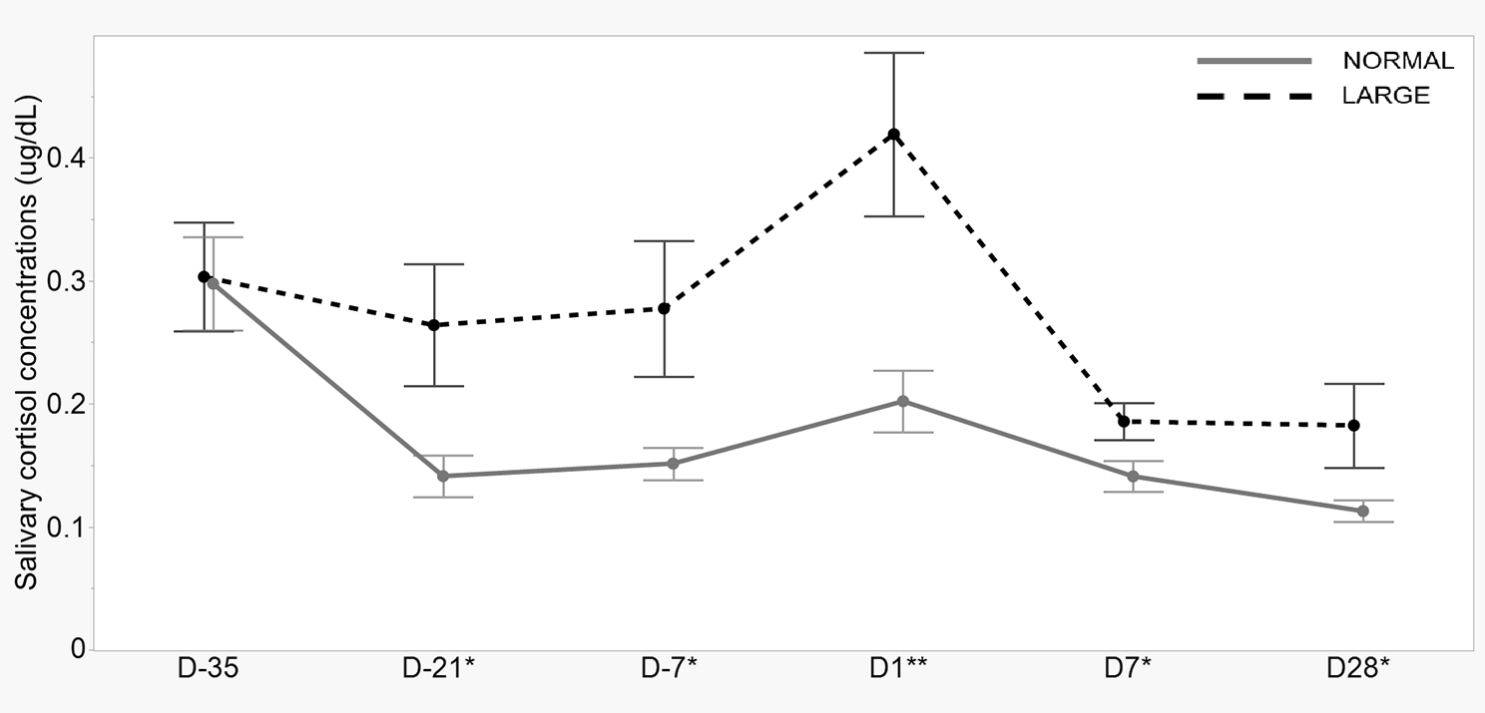
Salivary cortisol levels at 35, 21, and 7 d before parturition, and 1, 7, and 28 d after parturition between sows with normal litters (NORMAL, *n* = 8) and large litters (LARGE, *n* = 16). All salivary samples were collected around 1000 h on each sampling day. Values represent LS means and SEM. *P < 0.10, **P < 0.05, ***P < 0.01

Sows in the LARGE group tended to have higher cortisol levels on D-21, D-7, D7, and D28 compared to those in the NORMAL group (*P* < 0.10 for all, Figure). On D1, the cortisol levels of sows in the LARGE group were significantly greater than those of sows in the NORMAL group (*P* < 0.05, Fig. 1).

**Fig. 2:**
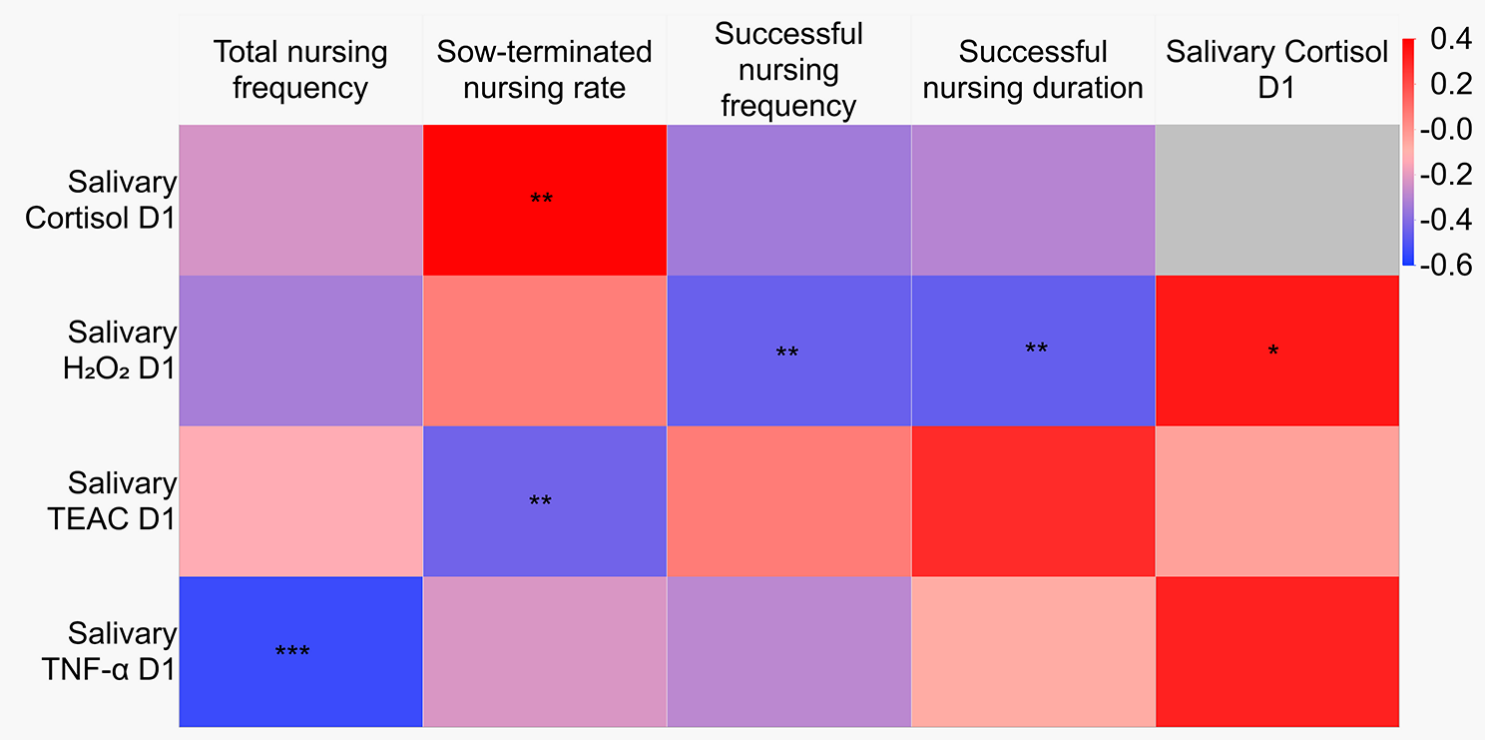
Spearman correlations between salivary oxidative stress parameters, salivary cortisol, and sow nursing behaviour. Data for salivary oxidative stress parameters were obtained from our previous study [13]. *P < 0.10, **P < 0.05, ***P < 0.01. Abbreviations: H_2_O_2_, Hydrogen peroxide; TEAC, Trolox equivalent antioxidant capacity; TNF-α, Tumour necrosis factor- α

There was a positive correlation between cortisol levels on D1 and the sow-terminated nursing rate (*r* = 0.42, *P* < 0.05, Fig. 2).

### Colostrum IgG, IgM, and IgA levels

There were no significant differences in the levels of IgG, IgM and IgA between the two groups (*P* > 0.10 for all, Table 3).

**Table 3:** Comparison of colostral IgG, IgM, and IgA levels between sows with normal litters (NORMAL) and large litters (LARGE)^1^

N	NORMAL	LARGE	SEM	*P* value
	8	16		
Concentrations (ng/ml)				
IgG	192.2	179.4	4.7	0.18
IgM	6.0	5.8	0.3	0.81
IgA	11.7	9.2	1.1	0.21

^1^ All colostrum samples were collected within 3 h after the initiation of parturition

### Correlations with oxidative stress parameters

The total nursing frequency was negatively correlated with levels of tumour necrosis factor-alpha (TNF-α) on D1 (*r* = − 0.54, *P* < 0.01, Fig. 2). Additionally, the sow-terminated nursing rate showed a negative correlation with Trolox equivalent antioxidant capacity (TEAC) levels on D1 (*r* = − 0.44, *P* < 0.05, Fig. 2), and the frequency of successful nursing exhibited a negative correlation with hydrogen peroxide (H_2_O_2_) levels on D1 (*r* = − 0.46, *P* < 0.05, Fig. 2). A significant negative correlation was also observed between the duration of successful nursing and H_2_O_2_ levels on D1 (*r* = − 0.47, *P* < 0.05, Fig. 2). On D1, cortisol levels showed a tendency towards a positive correlation with H_2_O_2_ levels (*r* = 0.35, *P* = 0.10).

When using litter size as a control variable, the sow-terminated nursing rate exhibited no statistically significant partial correlation with cortisol levels on D1 (*r* = 0.23, *P* > 0.10). Additionally, the partial Spearman correlation values using litter size as the control variable between successful nursing frequency and H_2_O_2_ levels on D1, as well as between H_2_O_2_ levels and cortisol levels on D1, did not yield statistically significant results (*r* = − 0.35, *P* > 0.10; *r* = 0.19, *P* > 0.10, respectively).

## Discussion

To the best of our knowledge, this is the first study to investigate the effects of litter size at the time of parturition on postpartum nursing and carefulness behaviour of sows. We confirmed that poor postpartum maternal behaviour and litter performance were correlated with increased physiological and oxidative stress levels induced by the large litter size of the farrowing sows. These findings emphasize the significance of addressing physiological and oxidative stress to achieve optimal lactation and litter performance in hyperprolific sows. In the meantime, performing cross-fostering within 2 d after birth and providing a milk replacer to all litters starting from 7 d after birth, as our primary focus was directed towards examining the physiological stress experienced by hyperprolific sows during the peripartum periods and its consequential impact on postpartum maternal behaviour and litter performance.

The current findings indicating poor litter performance in the LARGE group during early lactation are consistent with those of previous studies. These studies have shown that hyperprolific sows often exhibit poor litter performance due to insufficient milk production to support a large number of offspring [2, 18]. However, we did not observe any significant differences in litter performance at weaning between the two groups. This could be attributed to the specific care measures implemented in this study, such as cross-fostering and provision of milk replacers to all litters, under the management routine of the experimental farm. These measures were taken to prevent subpar litter performance caused by inadequate milk intake in large litters. In fact, recent studies reported that cross-fostering and the provision of milk replacer can prevent poor litter performance resulting from inadequate milk production and thereby improve piglet survival in large litters [19, 20]. Therefore, it is possible that these management strategies played a role in the absence of significant differences in litter performance at weaning between the NORMAL and LARGE groups.

We found greater postnatal piglet mortality rates due to crushing or other causes in large litters. This outcome may be linked to the reduced vitality of piglets in large litters. Our previous study by Lee et al. [13] confirmed that sows with large litters have longer farrowing durations. Prolonged farrowing, as confirmed in previous studies, can lead to neonatal piglet hypoxia, ultimately reducing piglet vitality [21]. Low-vitality piglets often encounter challenges in competing for teat access within large litters, leading to a reduction in colostrum intake. This diminished competitiveness for teat access contributes to piglet mortality attributed to various causes such as starvation and hypothermia [2, 22, 23]. Hypothermia, in particular, is closely associated with insufficient colostrum intake, as colostrum provides essential fats for thermogenesis [24, 25]. Moreover, it was reported that hypothermic piglets often seek warmth near the sow, making them more susceptible to being crushed [22, 24, 26, 27]. Baxter et al. [2] and Peltoniemi et al. [4] have also highlighted the association between poor colostrum intake and piglet deaths due to starvation, hypothermia, and crushing in hyperprolific sows. Therefore, the elevated postnatal piglet mortality rates observed in the LARGE group can be attributed to the limited colostrum intake of low-vitality piglets.

An observed tendency for increased piglet mortality rates was noted in large litters on D7, although the specific cause of death remained unconfirmed. Previous studies have indicated that piglet crushing is most prevalent within the first three days after birth, but hypothermia can contribute to piglet mortality not only during the neonatal period but also throughout the pre-weaning period [28, 29]. It is therefore plausible that low-vitality piglets with poor colostrum intake may experience long-term adverse effects on survival during the lactation period in large litters.

The present findings, which indicate poor nursing behaviour in large litters, can support the suggestion that deficient postpartum maternal behaviour in sows may be associated with physiological stress [30]. This stress can increase opioid receptors, inhibiting the secretion of oxytocin [7]. A previous study conducted on rats similarly found a negative correlation between corticosterone levels and the frequency of maternal care [31]. Consistent with these findings, the present study revealed a positive correlation between postpartum cortisol levels and the sow-terminated nursing rate. This implies that increased physiological stress may have a negative influence on nursing behaviour, possibly by affecting the secretion of maternal hormones.

Additionally, we identified negative correlations between the frequency of total nursing behaviour and postpartum TNF-α as well as between the frequency of successful nursing behaviour and postpartum H_2_O_2_ levels. Furthermore, the sow-terminated nursing rate exhibited a negative correlation with postpartum antioxidant capacity. It is important to note that oxidative stress inhibits the secretion of hormones that play a crucial role in promoting parental behaviour, such as oxytocin, vasopressin, and prolactin, which are produced via HPA axis activity [32–34]. Oxidative stress often accompanies physiological stress as it can result in the production of pro-inflammatory cytokines and reactive oxygen species through cell death [35–37]. This notion is substantiated by our finding with the partial correlation observed between postpartum cortisol and H_2_O_2_ levels. Furthermore, our companion study demonstrated that sows with larger litter sizes experience significant oxidative stress during the peripartum periods [13]. In this regard, the elevated levels of physiological and oxidative stress in sows in the LARGE group may have contributed to their suboptimal nursing behaviour.

In a recent study by Baxter et al. [30], the authors discovered that sows with high cortisol levels exhibited a reduced level of attentiveness towards their offspring when changing their posture. Our findings also revealed that sows in the LARGE group exhibited decreased careful behaviour when transitioning from standing to lying down. This finding implies that the pronounced stress experienced by sows in the LARGE group may contribute to a decline in their overall carefulness, as such attentiveness is known to be primarily regulated by oxytocin levels in sows [38, 39]. Consequently, this reduced carefulness in sows in the LARGE group may explain the elevated rates of postpartum piglet crushing compared to that in the NORMAL group.

Similar to a previous study by Roelofs et al. [12], which found a positive correlation between litter size at birth and cortisol concentrations during the last week of pregnancy in sows, the current findings provide evidence that sows with large litters experience higher stress levels compared to those with normal litter sizes. This elevated stress in sows with large litters appears to be primarily due to physiological and physical challenges. These challenges include the substantial energy requirements for developing numerous foetuses and limitations in movement caused by their larger body size [12, 40, 41]. Similarly, research on mice has shown that mice with large litters exhibit more anxiety-related behaviours during pregnancy, such as stereotyped climbing and maternal aggression, as a stress response [42, 43]. During the lactation period, sows also face various stressors, including increased energy demands for milk production and heat stress due to heightened metabolic activity [2, 44]. Therefore, our findings suggest that the physiological burdens and physical discomfort associated with large litter size can lead to increased cortisol levels in sows with large litters during late gestation. Additionally, it is probable that this elevated cortisol level likely persists until weaning, driven by the ongoing physiological challenges of milk production. However, it is noteworthy that the current findings suggest tendencies rather than statistical significance.

Mild stress triggers the secretion of glucocorticoids within the HPA axis, consequently leading to an increased synthesis of immunoglobulin [35, 45]. Conversely, severe and chronic stress can potentially impair immune system functionality and suppress the production of immunoglobulin [46]. Notably, stress is intricately related to oxidative stress, as stressors stimulate the production of pro-inflammatory cytokines that generate reactive oxygen species [35–37]. This oxidative stress negatively affects immune function by causing damage to the organs comprising the immune system, particularly in chickens [47]. Ercal et al. [17] suggested that oxidative stress induces immunosuppression, resulting in a decrease in serum immunoglobulin levels in rats. In our study, however, the large litter size does not appear to influence colostral immunoglobulin levels, which contradicts our initial hypothesis that such levels would vary in response to the increased stress and oxidative stress caused by a large litter. This may be because parturition itself is a powerful acute stressor that causes significant pain in sows, as reported in studies by Lawrence et al. [48], Mainau and Manteca [49], and Nagel et al. [50]. Therefore, we speculate that the parturition process acts as a severe acute stressor for sows in both NORMAL and LARGE groups, regardless of litter size. This stressor potentially leads to a decrease in colostral immunoglobulin levels in both groups, even though sows in the NORMAL group may experience lower stress levels, attributable to a comparatively lower number of foetuses, than those experienced by sows in the LARGE group during late gestation and parturition.

## Conclusions

In conclusion, this study highlights the negative impact of increased litter size on both litter performance and postpartum maternal behaviour in sows. The findings suggest that the compromised physiological status of sows is intricately associated with suboptimal postpartum maternal behaviour, which ultimately leads to increased piglet mortality during lactation. Therefore, in order to achieve optimum litter performance in the sows with large litters, it is imperative to formulate nutritional and management strategies aimed at mitigating potential increases in physiological and oxidative stress induced by the increased litter size in periparturient sows.

## Methods

The experimental protocols were approved by the animal experimental ethics committee of Chonnam National University (Approval number: 202106-189). The study was conducted on a commercial pig farm located in Southern South Korea from January to March 2022.

### Animals and housing

A total of 24 primiparous sows (Landrace$\times$Large white) were selected from a pool of 28 sows within a single farrowing batch. Detailed information regarding the experimental animals and farrowing housing conditions can be found in Lee et al. [13]. Briefly, the sows were divided into two groups based on litter size. The NORMAL group consisted of 8 sows with a litter size ranging from 7 to 14, while the LARGE group consisted of 16 sows with a litter size between 15 and 20. The LARGE group was specifically characterized by a litter size surpassing the number of functional teats on the sow, which was determined to be 14. Within 48 h after birth, the piglets from the LARGE group were cross-fostered exclusively to sows in the NORMAL group to ensure an equal number of piglets per sow. By performing cross-fostering, we controlled for the potential confounding effects of the increased number of suckling piglets on lactation performance by standardizing the number of piglets within 2 d after birth. This adjustment resulted in an average of 12.6 $\pm$ 2.3 piglets per sow. The piglets had free access to water through a water cup, and starting from 7 d after birth, a milk replacer was provided ad libitum to all piglets through a milk feeder (Mini Transition Feeder; Baker Feeders, UK). At day 28 ± 2 of lactation, all piglets were weaned.

### Data collection

#### Piglet mortality and body weight

Piglet mortality and body weight were assessed at three time points on the farm: day 1 (D1), day 7 (D7), and day 28 (D28). On D1, piglet mortality was categorized into two groups: (1) crushing, or (2) other causes, based on visible signs of injury such as bruises or broken bones.

#### Behavioural observation

All sows and piglets in the experiment were video-recorded from 72 to 96 h after the initiation of parturition to evaluate nursing and carefulness behaviours. Internet protocol (IP) cameras (HN0-E60; Hanwha Techwin, South Korea) were positioned 1.5 m vertically above their heads so that there was no blind spot. Per each two sows, the camera was installed. The sequence output was recorded using IP-camera software (Smart Cam Lite v.1.3.2, Hanwha Techwin, USA). Two trained researchers reviewed the recorded sequence output using a media player (Naver series on player, Naver Corp., South Korea). The display resolution was 1920 × 1080 pixels, and the frame rate was 30 FPS. One researcher observed half of the NORMAL and LARGE groups, and the other researcher observed the remaining NORMAL and LARGE groups. The researchers were blinded to the assignment of sows to the LARGE or NORMAL groups during video observations. Inter-observer reliability (IOR) was evaluated using the Cronbach alpha reliability test, yielding a substantial value of 0.97, indicating a significantly high level of agreement.

Nursing behaviour criteria were established following the procedure described by Valros et al. [51] and Yun et al. [52] (Table 4). The variables analysed included total and successful nursing frequency, duration and bout duration, as well as the termination of total nursing during a 24-h period (Table 4). The total nursing variables represent the combined sum of successful and unsuccessful nursing variables (Table 4).

**Table 4:** Definition and description of nursing and carefulness behaviours of sows

Variables	Definition
Nursing behaviour^1^	
Start of nursing	Over 50% of the piglets in a litter were vigorously massaging the udder
End of nursing	Over 50% of the piglets had either moved away from the udder or were in a state of inactivity close to the udder
Successful nursing	Piglets exhibited sucking behaviour that lasted for over 15 s following vigorous udder massaging
Unsuccessful nursing	Piglets did not show sucking behaviour
Sow-terminated nursing	When the sows were standing up or turning around to prevent further udder massage by the piglets
Carefulness behaviour^2^	
Sniffing	Sow uses her snout to touch the piglets before settling down
Rooting or Pawing	Sow uses her nose or front paws to dig into the floor before lying down
Turning the head towards piglets	Sow watches the position of the piglets while sitting, kneeling, or lying down
Progressing carefully	Careful, gentle, cautious, and slow movements while sitting, kneeling, or lying down
No piglets in the danger zone	There are no piglets positioned where the sows sit, kneel, or lie down

^1^The definition of nursing behaviour was adapted from Valros et al. [50] and Yun et al. [51]

^2^The definition of carefulness behaviour was adapted from Yun et al. [51]

To assess the carefulness behaviour of the sows, two parameters were utilized: the frequency of lying-down events, which is the number of times a sow transitions from a standing to a lying position, and the carefulness score during a 24-h period (Table 4). The carefulness score was calculated by adding up the occurrence of five behaviour patterns associated with sow carefulness while lying down. Each observed behaviour pattern was assigned a value of 1 if present or 0 if not present (Table 4, Yun et al. [52]).

#### Sow saliva sampling and assays

Saliva samples were obtained without restraint from each sow using synthetic swabs (Salivette^®^ Cortisol; Sarstedt, Nümbrecht, Germany) on days 35, 21, and 7 before farrowing (D-35, D-21, and D-7, respectively), and on days 1, 7, and 28 after farrowing (D1, D7, and D28, respectively), approximately 30 min before morning feeding (1030 h). The swabs were carefully placed and positioned around the back teeth using curved forceps until fully saturated, which typically took approximately 1 min. Immediately after collecting the saliva samples, they were stored at − 55 °C using a deep freezer on the farm during the sampling period. Subsequently, the saliva samples were transported to the laboratory while maintaining a temperature of − 20 °C using a portable freezer. In the laboratory, the samples were centrifuged for 15 min at 1500 rpm to analyse the cortisol levels. The resulting supernatant was allocated into pre-labelled 1.5-mL microcentrifuge tubes and stored at − 80 °C until further analysis.

To analyse salivary cortisol levels, an ELISA kit (Salimetrics Cortisol Enzyme Immunoassay Kit, Salimetrics, LLC., San Diego, USA) was used. No dilution was applied, and the manufacturer’s instructions were followed. The detection range of the kit was 0.012$$ -$$3.0 ug/dL. Both intra- and inter-assay CVs were below 10%.

The levels of salivary TNF-α, TEAC, and H_2_O_2_, were also determined using ELISA kits (MybioSource). For a more comprehensive understanding of the ELISA kits and their associated detection ranges, readers are directed to the detailed descriptions presented in our companion study by Lee et al. [13].

#### Colostrum sampling and assays

Approximately 30 mL of colostrum samples were obtained without restraint from each functional teat of all sows using a 50-mL conical tube (SPL, Pocheon, South Korea) ranging from the initiation of the parturition to after three hours. The teat order was not considered to minimize the colostrum sampling time required. During the sampling period, the collected colostrum samples were immediately frozen at − 55 °C using a deep freezer on the farm. Subsequently, the colostrum samples were transported to the laboratory while keeping − 20 °C through the use of a portable freezer. In the lab, the colostrum samples were centrifuged at 1000 × *g* for 15 min, and the aqueous fraction was collected. This process was repeated three times. The collected aqueous fraction samples were then stored at − 80 °C using a deep freezer until further analysis of IgG, IgM, and IgA.

Colostrum IgG, IgM and IgA levels were quantified using ELISA kits (Bethyl, E101-104; E101-117; E101-102, USA, respectively). Following the provided instructions, the aqueous fraction samples were diluted at ratios of 1:1,000,000, 1:50,000, and 1:30,000 in dilution buffer for IgG, IgM, and IgA analysis, respectively, and subsequently subjected to analysis. The detection ranges for IgG, IgM, and IgA were 1.37$$ -$$1000 ng/mL. The intra assay CVs for colostrum IgG, IgM, and IgA were below 10%.

### Calculations and statistical analysis

SAS version 9.4 (SAS Institute Inc., Cary, NC, USA) was used for the statistical processing of all data using a completely randomized design, where sow groups (LARGE and NORMAL) were treated as a fixed effect. The experimental unit was either the sow or the litter, and the results are presented as means with the standard error of the mean (SEM) indicated. The normality of the data was assessed using PROC UNIVARIATE with the Shapiro-Wilk test.

We used a PROC GLIMMIX model with a Lognormal distribution to determine the piglet mortality rate. Additionally, the PROC GLM model was fitted to the data for piglet body weight. A PROC GLIMMIX model with a Poisson distribution was also utilized to analyse the data for the frequency, duration and bout duration of total and successful nursing, and the sow-terminated nursing rate of total nursing behaviour. Salivary cortisol levels on D-35, D-21, D-7, D1, D7, and D28 were analysed using a PROC GLM model for repeated measures with a compound symmetry structure. The smallest AIC and BIC values were adopted to select the best model for salivary cortisol data analysis.

Furthermore, a PROC GLIMMIX model with a lognormal distribution was employed to analyse colostrum IgG, IgM, and IgA levels. In all PROC GLIMMIX models, a random statement was incorporated with a residual option.

To examine the correlations between nursing behaviour and cortisol, nursing behaviour and oxidative stress parameters, and cortisol and oxidative stress parameters, Spearman’s rank correlation coefficients (*r*) were used. Additionally, we conducted partial Spearman’s rank correlations where litter size served as the control variable to assess its impact on these relationships. Cortisol and oxidative data analysed using repeated measures throughout the entire sampling period were used. The data for oxidative stress parameters were obtained from our previous study [13].

## Data Availability

No datasets were generated or analysed during the current study.

## Abbreviations

IgG: Immunoglobulin G
IgM: Immunoglobulin M
IgA: Immunoglobulin A
SEM: Standard error of the mean
TNF-α: Tumour necrosis factor-alpha
TEAC: Trolox equivalent antioxidant capacity
H_2_O_2_: Hydrogen peroxide

## References

[1] Peltoniemi O, Yun J, Björkman S, Han T (2021). Coping with large litters: the management of neonatal piglets and sow reproduction. J Anim Sci Technol.

[2] Baxter EM, Schmitt O, Pedersen LJ. Managing the litter from hyperprolific sows. In the suckling and weaned piglet. Wageningen Acad Publishers. 2020; 347–56.

[3] Oliviero C, Junnikkala S, Peltoniemi O (2019). The challenge of large litters on the immune system of the sow and the piglets. Reprod Domest Anim.

[4] Peltoniemi O, Oliviero C, Yun J, Grahofer A, Björkman (2020). Management practices to optimize the parturition process in the hyperprolific sow. J Anim Sci.

[5] Farmer C (2001). The role of prolactin for mammogenesis and galactopoiesis in swine. Livest Prod Sci.

[6] Valros A, Rundgren M, Špinka M, Saloniemi H, Hultén F, Uvnäs-Moberg K, Tománek M, Krejcı P, Algers B (2004). Oxytocin, prolactin and somatostatin in lactating sows: associations with mobilisation of body resources and maternal behaviour. Livest Prod Sci.

[7] Algers B, Uvnäs-Moberg K (2007). Maternal behavior in pigs. Horm Behav.

[8] Van Vugt DA, Meites J (1980). Influence of endogenous opiates on anterior pituitary function. Fed Proc.

[9] Ragavan V, Frantz AG (1981). Opioid regulation of prolactin secretion: evidence for a specific role of β-endorphin. Endocrinology.

[10] Zanella AJ, Broom DM, Hunter JC, Mendl MT (1996). Brain opioid receptors in relation to stereotypies, inactivity, and housing in sows. Physiol Behav.

[11] Oliviero C, Heinonen M, Valros A, Hälli O, Peltoniemi OAT (2008). Effect of the environment on the physiology of the sow during late pregnancy, farrowing and early lactation. Anim Reprod Sci.

[12] Roelofs S, Godding L, de Haan JR, van der Staay FJ, Nordquist RE (2019). Effects of parity and litter size on cortisol measures in commercially housed sows and their offspring. Physiol Behav.

[13] Lee J, Shin H, Jo J, Lee G, Yun J. Large litter size increases oxidative stress and adversely affects nest-building behavior and litter characteristics in primiparous sows. Front Vet Sci 2023; 10. 10.3389/fvets.2023.1219572 PMC10477666 37675077

[14] Rooke JA, Bland IM (2002). The acquisition of passive immunity in the new-born piglet. Livest Prod Sci.

[15] Bourne FJ (1973). The immunoglobulin system of the suckling pig. Proc Nutr Soc.

[16] Machado-Neto R, Graves CN, Curtis SE (1987). Immunoglobulins in piglets from sows heat-stressed prepartum. J Anim Sci.

[17] Ercal N, Neal R, Treeratphan P, Lutz PM, Hammond TC, Dennery PA, Spitz DR (2000). A role for oxidative stress in suppressing serum immunoglobulin levels in lead-exposed Fisher 344 rats. Arch Environ Contam Toxicol.

[18] Špinka M, Illmann G. Nursing behavior. In the gestating and lactating sow. Wageningen Acad Publishers. 2015; 149–50.

[19] Peltoniemi O, Han T, Yun J (2021). Coping with large litters: management effects on welfare and nursing capacity of the sow. J Anim Sci Technol.

[20] Hoang QN (2023). Management strategies for sows and piglets to increase the Newborn piglets’ survivability rate. J Adv Vet Res.

[21] Herpin P, Le Dividich J, Hulin JC, Fillaut M, De Marco F, Bertin R (1996). Effects of the level of asphyxia during delivery on viability at birth and early postnatal vitality of newborn pigs. J Anim Sci.

[22] Baxter EM, Jarvis S, D’eath RB, Ross DW, Robson SK, Farish M, Nevison IM, Lawrence AB, Edwards SA (2008). Investigating the behavioural and physiological indicators of neonatal survival in pigs. Theriogenology.

[23] Heuß EM, Pröll-Cornelissen MJ, Neuhoff C, Tholen E (2019). Große-Brinkhaus, C. Invited review: piglet survival: benefits of the immunocompetence. Animal.

[24] Herpin P, Damon M, Le Dividich J (2002). Development of thermoregulation and neonatal survival in pigs. Livest Prod Sci.

[25] Uddin MK, Hasan S, Peltoniemi O, Oliviero C (2022). The effect of piglet vitality, birth order, and blood lactate on the piglet growth performances and preweaning survival. Porc Health Manag.

[26] Weber R, Keil NM, Fehr M, Horat R (2009). Factors affecting piglet mortality in loose farrowing systems on commercial farms. Livest Sci.

[27] Edwards SA, Baxter EM. Piglet mortality: causes and prevention. The gestating and lactating sow. Wageningen Academic Publishers; 2015. pp. 649–53.

[28] Sadeghi E, Kappers C, Chiumento A, Derks M, Havinga P. Improving piglets health and well-being: a review of piglets health indicators and related sensing technologies. Smart Agricultural Technology. 2023; 100246.

[29] Wang C, Han Q, Liu R, Ji W, Bi Y, Wen P, Yi R, Zhao P, Bao J, Liu H (2020). Equipping farrowing pens with straw improves maternal behavior and physiology of min-pig hybrid sows. Animals.

[30] Baxter EM, Hall SA, Farish M, Donbavand J, Brims M, Jack M, Lawrence AB (2023). Camerlink, I. Piglets’ behaviour and performance in relation to sow characteristics. Animal.

[31] Francis DD, Meaney MJ (1999). Maternal care and the development of stress responses. Curr Opin Neurobiol.

[32] Pantić VR (1995). Biology of hypothalamic neurons and pituitary cells. Int Rev Cytol.

[33] Ortiga-Carvalho TM, Chiamolera MI, Pazos‐Moura CC, Wondisford FE (2011). Hypothalamus‐pituitary‐thyroid axis Compr Physiol.

[34] Vitale G, Salvioli S, Franceschi C (2013). Oxidative stress and the ageing endocrine system. Nat Rev Endocrinol.

[35] Maes M, Hendriks D, Van Gastel A, Demedts P, Wauters A, Neels H, Janca A, Scharpé S (1997). Effects of psychological stress on serum immunoglobulin, complement and acute phase protein concentrations in normal volunteers. Psychoneuroendocrinology.

[36] Wang Y, Walsh SW (1998). Placental mitochondria as a source of oxidative stress in pre-eclampsia. Placenta.

[37] Volpe CMO, Villar-Delfino PH, Anjos D, Nogueira-Machado PMF (2018). Cellular death, reactive oxygen species (ROS) and diabetic complications. Cell Death Dis.

[38] Yun J, Swan KM, Farmer C, Oliviero C, Peltoniemi O, Valros A (2014). Prepartum nest-building has an impact on postpartum nursing performance and maternal behaviour in early lactating sows. Appl Anim Behav Sci.

[39] Ross HE, Young LJ (2009). Oxytocin and the neural mechanisms regulating social cognition and affiliative behaviour. Front Neuroendocrinol.

[40] Rutherford KMD, Baxter EM, D’eath RB, Turner SP, Arnott G, Roehe R, Ask B, Sandøe P, Moustsen VA, Thorup F, Edwards SA (2013). The welfare implications of large litter size in the domestic pig I: biological factors. Anim Welf.

[41] Seoane S, De Palo P, Lorenzo JM, Maggiolino A, González P, Pérez-Ciria L, Latorre MA (2020). Effect of increasing dietary aminoacid concentration in late gestation on body condition and reproductive performance of hyperprolific sows. Animals.

[42] Meek LR, Dittel PL, Sheehan MC, Chan JY, Kjolhaug SR (2001). Effects of stress during pregnancy on maternal behavior in mice. Physiol Behav.

[43] D’Amato FR, Rizzi R, Moles A (2006). Aggression and anxiety in pregnant mice are modulated by offspring characteristics. Anim Behav.

[44] Williams AM, Safranski TJ, Spiers DE, Eichen PA, Coate EA, Lucy MC (2013). Effects of a controlled heat stress during late gestation, lactation, and after weaning on thermoregulation, metabolism, and reproduction of primiparous sows. J Anim Sci.

[45] Campos-Rodríguez R, Godínez-Victoria M, Abarca-Rojano E, Pacheco-Yépez J, Reyna-Garfias H, Barbosa-Cabrera RE, Drago-Serrano ME (2013). Stress modulates intestinal secretory immunoglobulin A. Front Integr Neurosci.

[46] Cohen S, Miller GE, Rabin BS (2001). Psychological stress and antibody response to immunization: a critical review of the human literature. Psychosom Med.

[47] Zhang ZW, Wang QH, Zhang JL, Li S, Wang XL, Xu SW (2012). Effects of oxidative stress on immunosuppression induced by selenium deficiency in chickens. Biol Trace Elem Res.

[48] Lawrence AB, McLean KA, Jarvis S, Gilbert CL, Petherick JC (1997). Stress and parturition in the pig. Reprod Domest Anim.

[49] Mainau E, Manteca X (2011). Pain and discomfort caused by parturition in cows and sows. Appl Anim Behav Sci.

[50] Nagel C, Aurich C, Aurich J (2019). Stress effects on the regulation of parturition in different domestic animal species. Anim Reprod Sci.

[51] Valros AE, Rundgren M, Špinka M, Saloniemi H, Rydhmer L, Algers B (2002). Nursing behaviour of sows during 5 weeks lactation and effects on piglet growth. Appl Anim Behav Sci.

[52] Yun J, Swan KM, Vienola K, Farmer C, Oliviero C, Peltoniemi O, Valros A (2013). Nest-building in sows: effects of farrowing housing on hormonal modulation of maternal characteristics. Appl Anim Behav Sci.

